# Comparison of pharmacokinetic parameters of ranolazine between diabetic and non-diabetic rats 

**DOI:** 10.22038/IJBMS.2022.64391.14156

**Published:** 2022-07

**Authors:** Habibeh Mashayekhi-sardoo, Hossein Kamali, Soghra Mehri, Amirhossein Sahebkar, Mohsen Imenshahidi, Amir Hooshang Mohammadpour

**Affiliations:** 1 Department of Pharmacodynamics and Toxicology, School of Pharmacy, Mashhad University of Medical Sciences, Mashhad, Iran; 2 Targeted Drug Delivery Research Center, Pharmaceutical Technology Institute, Mashhad University of Medical Sciences, Mashhad, Iran; 3 Department of Pharmaceutics, School of Pharmacy, Mashhad University of Medical Sciences, Mashhad, Iran; 4 Biotechnology Research Center, Pharmaceutical Technology Institute, Mashhad University of Medical Sciences, Mashhad, Iran; 5 Applied Biomedical Research Center, Mashhad University of Medical Sciences, Mashhad, Iran; 6 Department of Biotechnology, School of Pharmacy, Mashhad University of Medical Sciences, Mashhad, Iran; 7 Pharmaceutical Research Center, Pharmaceutical Technology Institute, Mashhad University of Medical Sciences, Mashhad, Iran; 8 Department of Clinical Pharmacy, School of Pharmacy, Mashhad University of Medical Sciences, Mashhad, Iran

**Keywords:** Clearance, Diabetes mellitus, Pharmacokinetics, Ranolazine, Volume of distribution

## Abstract

**Objective(s):**

Diabetes mellitus (DM) affects the pharmacokinetics of drugs. Ranolazine is an antianginal drug that is prescribed in DM patients with angina. We decided to evaluate the effect of DM on the pharmacokinetics of ranolazine and its major metabolite CVT-2738 in rats.

**Materials and Methods:**

Male rats were divided into two groups: DM (induced by 55 mg/kg Streptozotocin (STZ)) and non-DM. All animals were treated with 80 mg/kg of ranolazine for 7 continuous days. The blood samples were collected immediately at 0 (prior to dosing), 1, 2, 3, 4, 8, and 12 hr after administration of the 7th dose of ranolazine. Serum ranolazine and CVT-2738 concentrations were determined using the high-performance liquid chromatography (HPLC) method. Pharmacokinetic parameters were calculated using a non-compartmental model and compared between the two groups.

**Results:**

The peak serum concentration (Cmax) and area under the curve (AUC) of ranolazine significantly decreased in DM compared with non-DM rats. DM rats showed significantly higher volumes of distribution (Vd) and clearance (CL) of ranolazine than non-DM rats. DM did not affect Ke, Tmax, and T1/2 of ranolazine. The concentration of metabolite was lower than the HPLC limit of detection (LOD).

**Conclusion:**

It was found that streptozotocin-induced DM increased Vd and CL of ranolazine, thereby decreasing the AUC of the drug. Therefore, dosage adjustment may be necessary for DM patients, which requires further clinical studies.

## Introduction

Diabetes mellitus (DM) as a growing healthcare challenge can affect the pharmacokinetics and pharmacodynamics of medications (1-3). Previous reports have shown that DM alters several physiological processes in humans, including micro-and macro vasculature, gastric mucosal blood flow, parasympathetic function, and intestinal hormone activity, which can affect the absorption of drugs (4-6). Moreover, poorly controlled blood glucose can cause gastropathy and changes in gastrointestinal pH. It ultimately leads to delays in gastric emptying, alterations in the lipophilicity of drugs, and decreased drug absorption (6, 7). Plasma protein binding of various drugs can change due to the modification in glycosylation of proteins after exceeding the produced plasma free fatty acids in the DM state (8). DM decreases vascular permeability through microvascular disorders (8). Previous clinical and experimental studies have revealed that DM impacts the CYP450 enzyme activity, but the findings have been inconsistent and contradictory (1, 9). It has been reported that the expression and function of CYP3A4 are diminished in DM subjects (9). Nevertheless, experimental studies have reported the suppression of microsomal N‐demethylase action and raised or even reduced levels of CYP450 enzymes. DM affects metabolism mainly by mechanisms such as increased reactive oxygen species (ROS) and oxidative stress (10). By affecting angiotensin‐converting enzymes and micro‐ and macro vascular structure of the kidney, DM can alter the renal elimination of drugs. These changes may impair drug delivery to relevant tissues and contribute to nephropathy development (8, 11).

Ranolazine is an anti-ischemic and antianginal drug that is indicated for decreasing the symptoms of chronic stable angina pectoris, myocardial infarction, and mortality rate in DM patients with ischemic heart disease (12).

The oral bioavailability of ranolazine is 30–50% (13) and for Ranexa^®^ tablets reaches 76% (14). Maximum (or peak) plasma concentrations of ranolazine are normally reached within 1 hour. The steady-state concentrations will typically be achieved within 3 days after oral administration. Ranolazine binds to α-1-acid glycoprotein in the blood. Although the elimination half-life of pure ranolazine is 1.4 to 1.9 hr, for sustained-release tablet formulation (ranolazine SR) it is about 7 hr. This half-life prolongation is because of the flip-flop kinetics of the SR preparation (15). 

**Figure 1:**
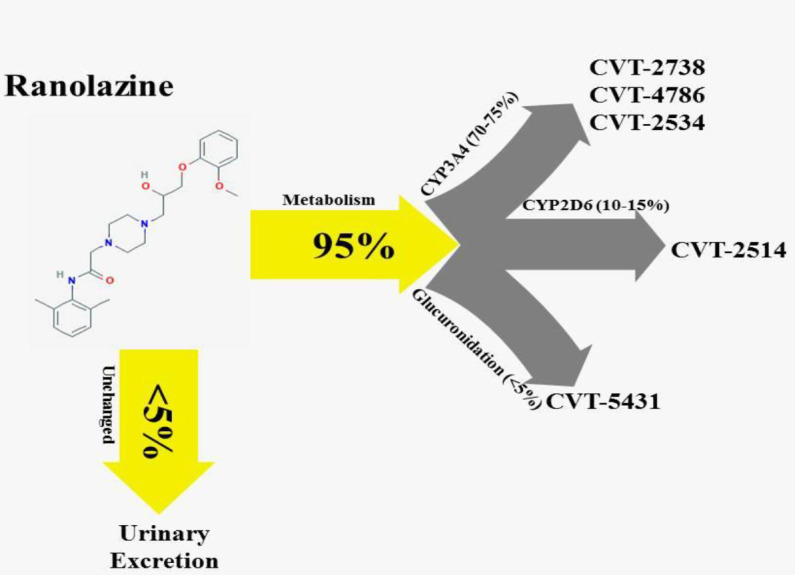
Metabolism pathway of ranolazine

Ranolazine is particularly metabolized by cytochrome P450 3A4 (CYP3A4; 70-75%) enzymes and to a lesser extent by CYP2D6 (less than 20%). The main metabolites of ranolazine are created through N-dealkylation of the piperidine ring (CVT-2738 and CVT-4786), O-demethylation (CVT-2514), methoxyphenoxy moiety O-dearylation (CVT-2512), and conjugation with glucuronide (CVT-5431). Consequently, 5% of ranolazine is excreted unaltered through the kidney (12, 16, 17) (Figure 1). Additionally, P-glycoprotein (P-gp) plays an important role in the reabsorption of ranolazine (18).

Since DM is known to affect the pharmacokinetics of drugs; the pharmacokinetics of ranolazine may also be altered in DM conditions, which will necessitate proper dose adjustment. For this reason, we decided to compare the pharmacokinetics of this drug and its metabolite CVT-2738 in DM and non-DM rats for the first time.

## Materials and Methods

Ranolazine (Ranexa® 500 mg, C_24_H_33_N_3_O_4_) was obtained from A. Menarini Pharma (UK) Company. The standard working of ranolazine and CVT-2738 (1-[(2,6-dimethylphenyl) aminocarbonylmethyl] Piperazine; C_14_H_21_N_3_O) was provided by Tinab Shimi Company (Mashhad, Iran). Acetonitrile gradient grade (CH₃CN, ≥ 99.9 % purity; CAT. No. 100030), Methanol gradient grade (CH₃OH, ≥ 99.9 %; CAT. No. 106007), Diethyl ether (C₄H₁₀O, ≥ 99.7 %; CAT. No. 100921), and Ortho-Phosphoric acid (H_3_PO_4_, 85%; CAT. No. 100573) were purchased from Merck Company (Rotexmeica, Germany). Potassium didrohygen phosphate (KH_2_PO_4_, >99.5%; CAT. No. CL00.1146) was obtained from Chem-Lab Company (Zedelgem, Belgium). Deionized water was used in the analytical experiment. Moreover, other solvents and chemicals used in the present study were of analytical grade. 

***Experimental animals***

Male rats (Wistar strain; 260–300 g) were ordered from Mashhad University of Medical Sciences, School of Pharmacy, Iran, Mashhad. The animals were housed in husk-filled polycarbonate plastic cages (25 ×15 × 40 cm), in a regular 24-hr interval and controlled light-dark cycle (12 hr-12 hr), temperature (21 ± 2 °C) circumstances. They freely had access to fresh water and a standard rodent chow diet (Behparvar Co, Karaj, Iran). 

*In vivo* *preclinical pharmacokinetic analyses*

The male Wistar rats were divided into two groups: DM and non-DM. On day 0, the non-DM group was treated with an intraperitoneal (IP) injection of normal saline; and DM animals after 10 hr fasting received an IP injection of 55 mg/kg STZ in normal saline (19). Plenty of water was available for the animals to prevent dehydration. The blood glucose at 72 hr after STZ injection was checked by Easy Gluco (Germany) and AccuCheck glucometer (Per forma, Roche, USA). Any animal having FBS over 250 mg/dl was considered diabetic (20).

The DM and non-DM rats since day 10 after STZ or normal saline injection received 80 mg/kg ranolazine (oral; once a day) dissolved in normal saline (freshly prepared daily) for 7 days. Blood samples were collected immediately 0 (prior to dosing), 1, 2, 3, 4, 8, and 12 hr after administration of the 7th dose of ranolazine. Six rats were used for every time point. The animals were sedated and the blood via retro-orbital plexus was collected. The serum was separated and stored at −80 °C until the extraction phase of ranolazine.

***HPLC method***

*Chromatographic conditions*

HPLC (A Shimadzu Prominence LC-20AD Liquid Chromatograph), an SPD-20A variable wavelength programmable UV/Vis detector, a CBM-20Alite system controller (Shimadzu Corporation, Gangnam-gu, Seoul, Korea). The analytical RP-C-18 column (Adamas® C18-Extreme; 5 µm, 250 mm x 4.6 mmID) was used in chromatography. The mobile phase consisted of phase A (phosphate buffer (pH = 2.2; 20 mM)) and phase B (acetonitrile); the pH was set up by ortho-phosphoric acid (1 mol/l). The gradient elution method was used as described in Table 1. The run time was adjusted to 16 min and the peak area of the samples was recorded by the Lab Solution Software. During the mobile phase preparation steps, the solvents were passed through a membrane filter and ultrasonically degassed before use. In all steps of the present experiment, the mobile phase was moved through the column at a flow rate of 1 ml/min and 40 °C. The UV detector at 214 nm was utilized. In each run, 20 µl of samples were injected.

*Method validation*

Linearity, precision, accuracy, the limit of detection (LOD), and limit of quantitation (LOQ) of the method were estimated according to the ICH Q2 (R1) guideline “validation and analytical procedures: text and methodology” (21). 

***Linearity***

To evaluate the linearity and construction of the calibration curve, eight-point calibration curves in the range of 4.875–312 ng/ml of ranolazine and 1250–20000 ng/ml of CVT-2738 were determined with triplicate measurement of each peak area of ranolazine and CVT-2738 in different concentrations (22).

***Limit of detection and limit of quantitation***

LOD and LOQ of the present analytical method were estimated according to 3.3:1 and 10:1 signal-to-noise ratio, respectively, by the following equations (23):

$\mathrm{LOD}=\frac{\mathrm{std y intercept}}{\mathrm{slope of calibration curve}}\times 3.3$

$\mathrm{LOQ}=\frac{\mathrm{std y intercept}}{\mathrm{slope of calibration curve}}\times 10$

***Accuracy and precision***

Accuracy was analyzed by measuring the percentage of recovery of three concentration levels of ranolazine and CVT-2738 (50%, 100%, and 150%) at five different times. Precision was also calculated by measuring the standard deviation at intra-day and inter-day five times (24).

*Sample preparation*

Concerning the serum extraction, 0.5 ml of each rat serum was added to 0.5 ml of diethyl ether and vortexed for 10 min and then was sonicated for 15 min (two times). After that, the content was centrifuged at 10000 rpm for 15 min at 4 °C (three times). The upper separated layer was collected and evaporated by nitrogen gas (18). The rat serum residues were immediately reconstituted in methanol and then injected into the HPLC system.

***Pharmacokinetic analysis***

The non-compartmental pharmacokinetic model was carried out using the Microsoft Excel add-in program PKSolver (25). The pharmacokinetic parameters including the area under the curve of serum concentration (AUC) versus time, the maximum concentration recorded (C_max_), the time taken to reach C_max_ (T_max_), the half-life of elimination (T½), the volume of distribution (V_d_), elimination rate (K_e_), and total drug clearance (CL) were determined (26).

***Statistical analysis***

The analytical data were expressed as mean ± SD. Statistical analyses were conducted using GraphPad Prism 8.0 (GraphPad Prism Software Inc., San Diego, USA). The significance was determined by the independent samples *t*-test. The findings were significant at *P*<0.05 level. 

## Results

***HPLC results***

**Figure 2:**
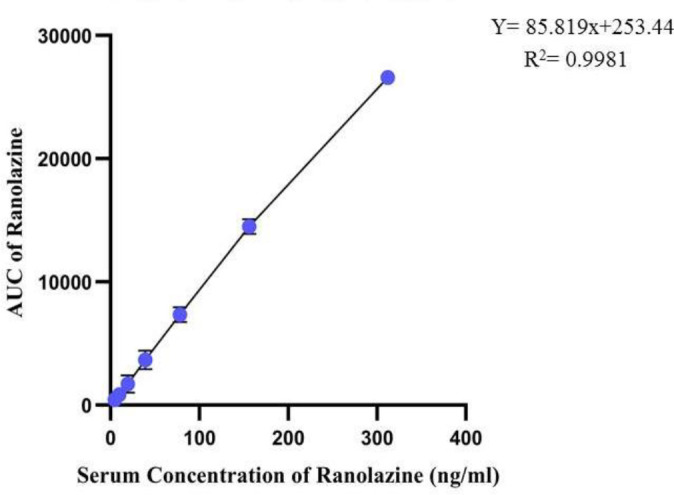
Calibration curve of ranolazine

**Figure 3:**
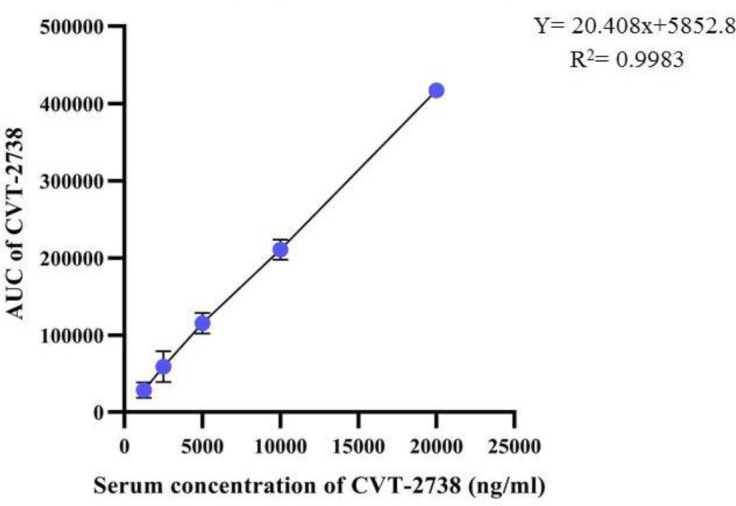
Calibration curve of CVT-2738

The findings of the chromatographic method validation of ranolazine and CVT-2738 were mean absolute recovery (91.8 and 92.4%; respectively); intra-and inter-day repeatability was less than 10% for serum concentrations. The standard curves represented excellent linearity for ranolazine and CVT-2738 with coefficients of correlation (r) more than 0.99 (Figures 2 and 3). The lower limits of detection (LOD) of ranolazine and CVT-2738 were 24.76 ng/ml and 940 ng/ml, respectively. The limits of quantitation (LOQ) of ranolazine and CVT-2738 were 51.59 ng/ml and 1200 ng/ml, respectively.

**Figure 4:**
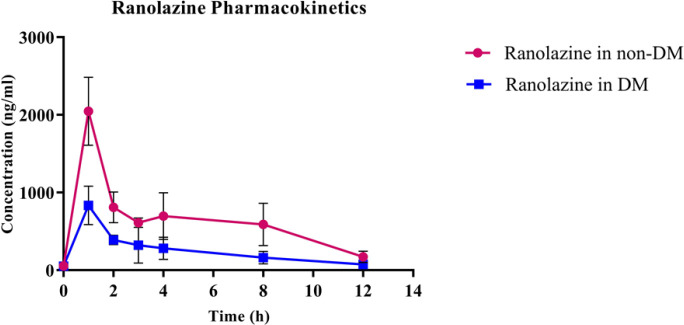
The pharmacokinetic profiles of ranolazine after oral administration in DM and non-DM groups of rats

The pharmacokinetic profile of ranolazine and its metabolite CVT-2738 was determined after the 7th administration of ranolazine (80 mg/kg b.w., oral) in normal saline to DM and non-DM rats. The serum concentration of ranolazine versus the time profile appears in Figure 4.

***Effects of DM on the absorption pharmacokinetic parameters of ranolazine (AUC, C***_max,_ ***and T***_max_ ***)***

The significantly lower AUC_0-12_ of ranolazine (3164.33 ± 501.87 ng/ml/min) was observed in DM compared with non-DM animals (8036.50 ± 2377.93 ng/ml/min) after oral administration of 80 mg/kg ranolazine (*P*<0.001).

The orally administered ranolazine showed significantly lower C_max_ (899.94 ± 387.85 ng/ml) in DM compared with non-DM rats (1911.25 ± 975.90 ng/ml; *P*<0.05). 

The maximum serum concentration of ranolazine was achieved in about 1 ± 0.00 hr after oral administration in DM and 1 ± 0.55 hr in non-DM animals, and the difference was not statistically significant (*P*>0.05).

***Effect of DM on the volume of distribution of ranolazine***

A higher V_d_ of ranolazine was observed in DM animals (133.70 ± 2.81 L/Kg) compared with non-DM animals (60.77 ± 2.32 L/Kg; *P*<0.001). 

***Effects of DM on clearance of ranolazine***

The CL of ranolazine in DM rats (21.80 ± 5.00 ng/ml/h) was significantly higher than in non-DM rats (9.91 ± 4.83 ng/ml/hr; *P*<0.01). There were no significant changes in K_e_ of ranolazine in DM and non-DM rats (*P*>0.05). 

The pharmacokinetic parameters of ranolazine were calculated and are presented in Table 2.

**Table 1:** The Gradient elution method for the determination of ranolazine and CVT-2738

Time (mean)	Module	Command	Value
0.01	Pumps	Solvent B	0
3	Pumps	Solvent B	40
8	Pumps	Solvent B	40
11	Pumps	Solvent B	100
14	Pumps	Solvent B	0
16	Pumps	Solvent B	0
16	Controller	Stop	-

The mobile phase consisted of phase A (phosphate buffer (pH = 2.2; 20 mM)) and phase B (acetonitrile)

**Table 2:** Steady-state pharmacokinetic parameters after oral (80 mg/kg) administration of ranolazine to DM and non-DM rats

Pharmacokinetic parameters	Non-DM rats	DM rats
AUC _0-12 _(ng/ml/h)	8036.50 ± 2377.93	3164.33 ± 501.87^***^
C_max_ (ng/ml)	1911.25 ± 975.90	899.94 ± 387.85^*^
T_max_ (h)	1 ± 0.55	1 ± 0.00
Half-life (h)	4.10 ± 1.93	4.83 ± 2.44
V_d _(L/kg)	60.77 ± 2.32	133.70 ± 2.81^***^
CL (ng/ml)/h	9.91 ± 4.83	21.80 ± 5.00^**^
K_el _(1/h)	0.16 ± 0.04	0.17 ±0.05

Values are expressed as means ± SD (n=6). Statistical independent samples t-test was used to assess the level of statistical differences between the groups: ***= *P*<0.001, **=*P*<0.01, and *=*P*<0.05 for comparison with non-DM animals DM: diabetes mellitus, Kel: elimination constant; half-life: time half-life; AUC 0-12: area under plasma concentration/time plot until the last quantifiable value; Cl: clearance; Vd: volume of distribution; Ka: absorption constant; Cmax: maximum concentration; Tmax: time to reach Cmax

## Discussion

Since ranolazine is prescribed in DM patients with angina (27), we decided to compare the pharmacokinetic profile of this drug and its metabolite CVT-2738 between DM and non-DM states. The findings indicated that DM significantly decreases AUC and C_max_ while it increases CL and V_d_ of this drug. Furthermore, DM did not affect K_e_, T_1/2, _and T_max_ of ranolazine.

In the present study, the AUC and C_max_ of ranolazine in DM animals were significantly lower than in non-DM animals. We suggest that DM as a P-gp expression inducer with no effect on metabolism (28) is able to reduce the AUC of ranolazine. Alfarisi *et al.* (29) reported a decline in plasma isoniazid and pyrazinamide concentrations in DM through several processes including elevation in intestinal motility, expression, and activity of P-gp. Adithan *et al*. (30) reported lower steady-state concentrations of phenytoin in patients with DM compared with controls. Nevertheless, the AUC of phlorizin was increased after down-regulation of P-gp and elevated intestinal tract permeability in DM rats (31).

Based on our previous study (32) showing inhibition of CYP3A2 in DM rats, we expected an increase in the serum concentration of ranolazine. However, in the current study, we observed that the increased V_d_ was an important factor influencing the pharmacokinetics of ranolazine, thereby decreasing its serum concentration. Different drugs have been shown with lower, equivalent, or higher distribution in DM versus non-DM conditions (33). DM can lead to an increase in V_d_ of drugs (34). Recently, Fediuk *et al*. (35) suggested that DM contributes to an enhancement in the apparent central volume of distribution (Vc/F) of ertugliflozin, thereby diminishing its C_max_ without any effect on the AUC value of the mentioned drug. Higher V_dss_ for torasemide and omeprazole in DM rats compared with control rats have also been reported (36). Patients with DM have increased V_d_ for paracetamol (acetaminophen) and theophylline (37, 38). In contrast, decreased V_1_ and V_dss_ of glimepiride in type 2-DM rats in comparison with those of the control and type 1-DM groups were reported (39). DM by glycosylation of plasma proteins reduces the binding of the drug to the relevant protein and increases the concentration of the free form of the drug in the blood. Therefore, the free drug can be distributed from plasma to the tissues, thereby increasing V_d_ (40). The V_d_ of lipophilic drugs may be affected by DM, and the increase in the lipophilicity of drugs is proportional to their V_d_ (41). Since ranolazine is a drug with high lipophilicity (42), we propose that DM has led to an increase in the distribution of this drug in the current study.

Considering that the K_e_ of ranolazine was similar in DM and non-DM groups, we assume that the elevated V_d_ of this lipophilic drug resulted in the increase of CL, thereby decreasing the AUC of ranolazine in DM animals. Apart from the fact that V_d_ can affect the CL of drugs, in the following, we will review studies regarding the effect of DM on the CL of drugs. DM predisposes to micro-and macrovascular disorders; hence it results in hyperfiltration and elevation in glomerular filtration rate. As shown in previous studies, a positive correlation exists between the doses of drugs and the glomerular filtration rate (28, 43). Through elevation of urine output, DM can increase the renal elimination of medications (44). Alteration of urine pH in DM conditions as a result of ketone production can affect urinary elimination of drugs (45). By speeding up the hepatic blood flow, DM increases the first-pass effect and then increases the hepatic CL, thereby increasing the total CL (46). A 3.4-fold increase in oral chlorzoxazone CL and then lower oral AUC were expressed in Type II-DM patients in comparison to healthy subjects (47). Likewise, after oral administration of clarithromycin to non-DM and DM rats, faster CL, thereby lower AUC of the mentioned drug in DM rats, was distinguished (48). Lee *et al.* in 2013 (49) reported similar urinary excretion of metoprolol in DM and non-DM rats. In contrast to our findings, DM rats exhibited lower CL but higher C_max_/AUC of paclitaxel compared with non-DM rats (50).

In the current study, we expected an increase in the serum concentration of ranolazine, but the increased V_d_ was an important factor influencing the pharmacokinetics of the drug. This increased the CL of ranolazine and thereby decreased its serum concentration. The present findings imply that Streptozotocin-Induced DM can affect the pharmacokinetic profile of ranolazine. This finding highlights the need for dose adjustment of ranolazine in DM patients after clinical research.

## Conclusion

The findings of our study indicated that increased V_d_ of ranolazine in DM rats resulted in the elevation of ranolazine CL and thereby a significant decrease in AUC and C_max_ of the drug. Further studies are required to explore the dosage adjustment of ranolazine in DM patients with chronic angina.

## Authors’ Contributions

HMS Performed acquisition, analysis, and drafting of the work. HK Helped with investigation, acquisition, and analysis. SM Helped write, review, and edit. AHS Proofread the paper. MI Reviewed and edited. AHM Designed the work and interpreted the data. HMS, HK, SM, AHS, MI, and AHM Approved the final version to be published.

## Ethics Approval

Ethical number: IR.MUMS.PHARMACY.REC.1397.051

## Conflicts of Interest

The authors state no conflicts of interest. 
